# Ultra-robust informational metasurfaces based on spatial coherence structures engineering

**DOI:** 10.1038/s41377-024-01485-3

**Published:** 2024-06-04

**Authors:** Leixin Liu, Wenwei Liu, Fei Wang, Xiaofeng Peng, Duk-Yong Choi, Hua Cheng, Yangjian Cai, Shuqi Chen

**Affiliations:** 1 Shandong Provincial Engineering and Technical Center of Light Manipulations, Collaborative Innovation Center of Light Manipulation and Applications, Shandong Provincial Key Laboratory of Optics and Photonic Device, School of Physics and Electronics, Shandong Normal University, Jinan, 250014 China; 2 The Key Laboratory of Weak Light Nonlinear Photonics, Ministry of Education, School of Physics, School of Materials Science and Engineering, Smart Sensing Interdisciplinary Science Center, Nankai University, Tianjin, 300071 China; 3 School of Physical Science and Technology, Soochow University, Suzhou, 215006 China; 4 Laser Physics Centre, Research School of Physics, Australian National University, Canberra, ACT 2601 Australia; 5 The Collaborative Innovation Center of Extreme Optics, Shanxi University, Taiyuan, Shanxi 030006 China

**Keywords:** Imaging and sensing, Metamaterials

## Abstract

Optical information transmission is vital in modern optics and photonics due to its concurrent and multi-dimensional nature, leading to tremendous applications such as optical microscopy, holography, and optical sensing. Conventional optical information transmission technologies suffer from bulky optical setup and information loss/crosstalk when meeting scatterers or obstacles in the light path. Here, we theoretically propose and experimentally realize the simultaneous manipulation of the coherence lengths and coherence structures of the light beams with the disordered metasurfaces. The ultra-robust optical information transmission and self-reconstruction can be realized by the generated partially coherent beam with modulated coherence structure even 93% of light is recklessly obstructed during light transmission, which brings new light to robust optical information transmission with a single metasurface. Our method provides a generic principle for the generalized coherence manipulation on the photonic platform and displays a variety of functionalities advancing capabilities in optical information transmission such as meta-holography and imaging in disordered and perturbative media.

## Abstract (web summary)

We propose a scheme for simultaneous manipulation of the coherence lengths and coherence structures of the light beams with the disordered metasurfaces, which further carry ultra-robust optical information.

## Graphical abstract

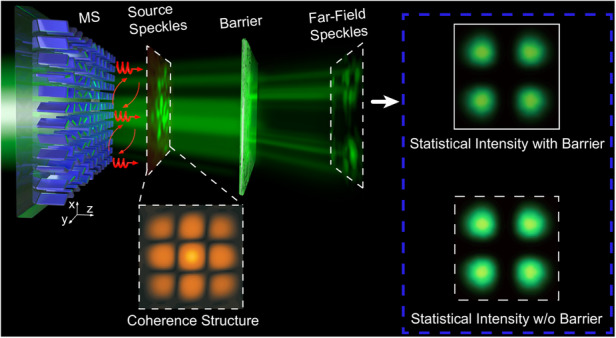

## Introduction

Information transmission plays a vital role in both theoretical physics and applied technology. In the past decades, optical information transmission has gathered great interest due to its concurrent and multi-dimensional nature^1,2^, leading to optical technologies such as super-resolution imaging^3^, fluorescent biological detection^4^, holography^5,6^, and optical computation^7^. However, optical information transmission is prone to loss and crosstalk when transmitting in inhomogeneous media containing scatterers or obstacles in the light path, especially for high informational spatial frequencies. Such information loss can be restored by employing a computational imaging scheme such as using the ghost imaging technology^8,9^, which can realize imaging using light that has never physically interacted with the object to be imaged. Recently, Klug et al. realized robust structured light transmitting as eigenmodes of atmospheric turbulence, which varies far slower than light traveling time^10^. These computational imaging methods usually suffer from complex and bulky optical setup, and the corresponding imaging technique can hardly be expanded to different imaging scenarios in a compatible manner, especially for radical disturbances. Researchers also endeavor to develop optical topological insulators to tackle scattering problems for robust optical transmission, but current schemes can hardly carry arbitrary patterned information. Besides, the operating media needs artificial modulation and it cannot operate in homogeneous media such as in free space^11–13^. Although artificial intelligence (AI) provides a routine for optical information transmission with improved robustness^14,15^, the limit of it still highly relies on the optical setup to obtain the original optical information.

Originating from the local or extended optical resonances that depend on both the constituent materials and geometric designs of the nanostructures, metasurfaces can achieve efficient optical manipulation from near-fields to far-fields^16,17^, which provide new opportunities to develop integrated optics and advanced photonics. They can not only realize compact optical manipulation but also provide a new perspective on flexibly shaping the light fields by manipulating their phase, amplitude as well as polarization at will via a compact and easy-of-fabrication system^18–20^. Owing to this versatility, various applications of metasurfaces have been proposed, including imaging^21,22^, invisibility^23^, holography^24,25^, multi-dimensional optical information transmission^26^, AI-aided reprogrammable imager^27,28^, etc. However, the conventional metasurface-based informational technologies usually suffer from severe information loss and crosstalk when meet disturbances, such as scatterers and obstacles in the optical path, partly owing to the ubiquitous diffraction effect resulting from the minimized metasurfaces, especially in the visible regime.

Here, taking advantage of the global correlation provided by spatial coherence manipulation^29–35^, we propose a scheme to achieve ultra-robust information transmission using disordered metasurface. With a random phase distribution to form a disorder-engineered waveform, the partially coherent beams with prescribed spatial coherence structures and coherence lengths, such as Hermite–Gaussian correlated Schell-model (HGCSM) beams and Laguerre-Gaussian correlated Schell-model (LGCSM) beams, are demonstrated. The coherence-structure-manipulated beam can exhibit extraordinary propagation properties such as the ultra-robust optical information transmission and the self-reconstruction effect, even though most of the light is recklessly obstructed. The proposed scheme paves the way for ultra-robust information propagation by engineering the spatial coherence structures and coherence lengths beyond conventional metasurface-based informational technologies.

## Results

### Principle of customizing spatial coherence structure and design strategy of the metasurface

Based on the optical statistics theory, the spatial coherence is a second-order statistical property describing the correlation between two spatial points of the random light fields. The characteristics of spatial coherence are calculated by the degree of coherence (DOC) function^36^:

1 $$\mu ({{\mathbf{\rho }}}_{1},{{\mathbf{\rho }}}_{2})=\frac{J({{\mathbf{\rho }}}_{1},{{\mathbf{\rho }}}_{2})}{\sqrt{J({{\mathbf{\rho }}}_{1},{{\mathbf{\rho }}}_{1})J({{\mathbf{\rho }}}_{2},{{\mathbf{\rho }}}_{2})}}$$

where ${{\mathbf{\rho }}}_{1}$ and ${{\mathbf{\rho }}}_{2}$ are two position vectors. $J({{\mathbf{\rho }}}_{1},{{\mathbf{\rho }}}_{2})=\langle {E}^{\ast }({{\mathbf{\rho }}}_{1})E({{\mathbf{\rho }}}_{2})\rangle$ is the mutual intensity function, where *E* denotes the random electric fields, the asterisk and angle brackets represent the complex conjugate and ensemble average over random fluctuations, respectively. The upper and lower limits of the modulus of the DOC function are 1 and 0, describing fully coherent and incoherent beams. For spatially uniform coherence of light, the DOC function depends only on the separation of two spatial points, i.e., $\mu ({{\mathbf{\rho }}}_{1},{{\mathbf{\rho }}}_{2})=\mu ({{\mathbf{\rho }}}_{2}-{{\mathbf{\rho }}}_{1})$. We consider a stochastic field generated by a deterministic beam passing through a random complex screen, given by $E({\mathbf{\rho }})=A({\mathbf{\rho }})T({\mathbf{\rho }})$, where $A({\mathbf{\rho }})$ and $T({\mathbf{\rho }})$ represent the complex deterministic electric fields and a complex screen, respectively. Taking the autocorrelation of $E({\mathbf{\rho }})$, we obtain the relation of the random phase screens and the DOC function as $\mu ({{\mathbf{\rho }}}_{2}-{{\mathbf{\rho }}}_{1})=\langle {T}^{\ast }({{\mathbf{\rho }}}_{1})T({{\mathbf{\rho }}}_{2})\rangle$^37^. It provides a way to generate random fields with customized spatial coherence structure by designing the complex screen $T({\mathbf{\rho }})$:

2 $$T({\mathbf{\rho }})=\int {\int _{-\infty }^{\infty }}r({\bf{f}}){[\frac{P({\bf{f}})}{2}]}^{1/2}\exp (i2\pi {\bf{f}}\cdot {\mathbf{\rho }}){d}^{2}f$$

where ${\bf{f}}=\hat{{\bf{x}}}{f}_{x}+\hat{{\bf{y}}}{f}_{y}$ is the spatial frequency vector, *r*(**f**) is a delta-correlated function composed of zero-mean and unit-variance complex Gaussian random numbers, and *P*(**f**) is the power spectral density (PSD) of the light beams, which is the Fourier transform of the DOC function. In practical applications, it is challenging to simultaneously modulate the random amplitude and phase. However, even if we only extract the phase part from the complex screen, i.e., $\varphi ({\mathbf{\rho }})=\angle T({\mathbf{\rho }})$, the autocorrelation of the pure phase screen is almost the same as that of the complex screen according to the simulations in Fig. S1 in Supplemental Material. Therefore, we use the random phase screen instead of the complex screen to customize the spatial coherence structure:

3 $$\langle \exp [-i\varphi ({{\mathbf{\rho }}}_{1})]\exp [i\varphi ({{\mathbf{\rho }}}_{2})]\rangle \approx \mu ({{\mathbf{\rho }}}_{2}-{{\mathbf{\rho }}}_{1})$$

The phase fluctuation of $\varphi ({\mathbf{\rho }})$ can be designed with a prescribed DOC $\mu ({{\mathbf{\rho }}}_{2}-{{\mathbf{\rho }}}_{1})$ to manipulate the coherence length and coherence structure of the incident beams.

**Fig. 1:**
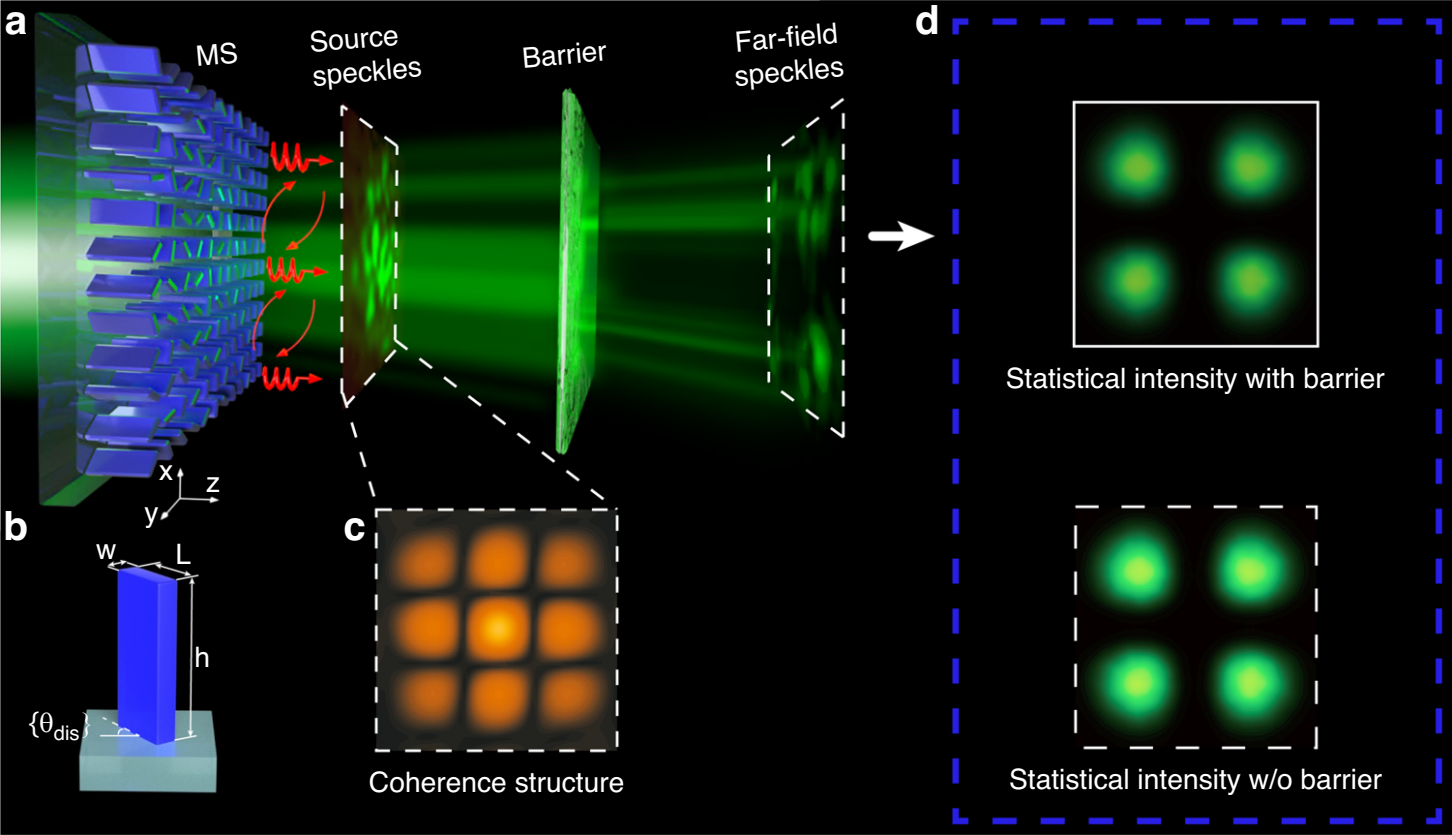
**Design of the disordered metasurface and its operation principle. a** The incident light passing through the disordered metasurface with a modified coherence structure and generating source speckles. With a scattering barrier blocking out the optical transmission, the far-field speckles are obtained. **b** The unit cell of the designed nanofin with disorder-engineered orientation set {θ_dis_}. **c** The coherence structure obtained from the source speckles in **a**. **d** The statistical intensity statistically obtained from the far-field speckles, containing the transmitted optical information with (top) and without (bottom) the barrier

With the ability of metasurfaces to accurately engineer the wave-front of light beams^38–40^, we project the phase fluctuation onto the metasurfaces to simultaneously realize coherence length and coherence structure manipulation. The spatial coherence structure distributions as an information carrier can be manipulated to realize robust optical information transmission. The schematic of the spatial coherence-manipulated design is shown in Fig. 1. A prescribed DOC with a random phase distribution and a specifically designed coherence structure is loaded onto the metasurface (Fig. 1a, c), resulting in a random distribution of source speckles during light propagation. The transmitted phase can be locally manipulated by large aspect-ratio TiO_2_ nanofins with a high refractive index and high transmittance. The phase distribution is achieved by the Pancharatnam-Berry phase following the formula *φ* (**r**) = 2σθ_dis_ (**r**), where σ = ±1 represents the left-/right-handed circularly polarized incident light, and θ_dis_ is the local orientation angle of the nanofins. The TiO_2_ nanofin has a length of L = 260 nm, a width of w = 90 nm, and a height of h = 550 nm located on the fused silica substrate with a lattice size of 330 nm (Fig. 1b). The operating wavelength is 532 nm. The statistical properties of the transmitted fields arise from the randomly arranged nanostructures of the phased array with prescribed correlation functions. The scattering or block barrier can significantly affect the light transmission and vary the wave-front of light beams. When superposing numerous instantaneous speckle intensities in the far-field, the statistical intensity is almost the same as that without the barrier (Fig. 1d), except for the inevitable loss of energy caused by the barrier.

**Fig. 2:**
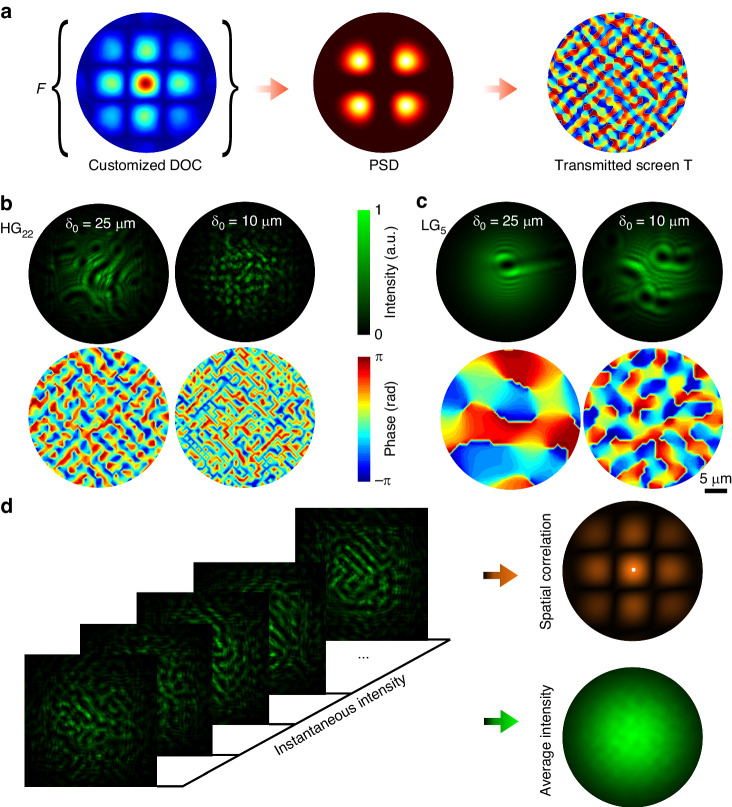
**Correlation between the DOC and the disordered metasurface design. a** Diagram of the synthesis of the transmitted function *T* of the disordered metasurface. **b**, **c** Intensity (top) and phase (bottom) distributions on the metasurfaces to manipulate spatial coherence with various prescribed DOC (HG_22_ in **b**, LG_5_ in **c**). The coherence lengths are 25 μm (left) and 10 μm (right). **d** An illustration of the spatial coherence manipulation with the metasurface. The spatial correlation of the instantaneous intensity distributions at the source plane determines the DOC of the average intensity transmitted from the metasurface

A proof-of-concept design to demonstrate the proposed strategy is shown in Fig. 2. To manipulate the spatial coherence structure and coherence length of the beams, we first investigate the mapping of the on-demand Schell-Model DOC to the phase distributions $\varphi ({\mathbf{\rho }})$ of the metasurface (Fig. 2a). A PSD can be calculated from the Fourier transform of the desired DOC. The phase distribution of the metasurface can be further calculated by the PSD from Eq. (2). We proposed two series of designs to generate beams with different spatial coherence structures and coherence lengths, i.e., Hermite–Gaussian correlated structure with *m* = 2, *n* = 2 (HG_22_) and Laguerre-Gaussian correlated structure with *n* = 5 (LG_5_), as shown in Fig. 2b, c. The DOC of the related HGCSM beams^41^ (Fig. 2b) and LGCSM beams^42^ (Fig. 2c) are defined as:

4 $$\begin{array}{c}{\mu }_{HG}({{\mathbf{\rho }}}_{1}-{{\mathbf{\rho }}}_{2})=\frac{{H}_{2m}[({x}_{1}-{x}_{2})/\sqrt{2}{\delta }_{0}]}{{H}_{2m}(0)}\exp [-\frac{{({x}_{1}-{x}_{2})}^{2}}{2{\delta }_{0}^{2}}]\\ \times \frac{{H}_{2n}[({y}_{1}-{y}_{2})/\sqrt{2}{\delta }_{0}]}{{H}_{2n}(0)}\exp [-\frac{{({y}_{1}-{y}_{2})}^{2}}{2{\delta }_{0}^{2}}]\end{array}$$

5 $$\begin{array}{c}{\mu }_{LG}({{\mathbf{\rho }}}_{1}-{{\mathbf{\rho }}}_{2})=\exp [-\frac{{({x}_{1}-{x}_{2})}^{2}}{2{\delta }_{0}^{2}}-\frac{{({y}_{1}-{y}_{2})}^{2}}{2{\delta }_{0}^{2}}]\\ \times {L}_{n}^{0}[\frac{{({x}_{1}-{x}_{2})}^{2}}{2{\delta }_{0}^{2}}+\frac{{({y}_{1}-{y}_{2})}^{2}}{2{\delta }_{0}^{2}}]\end{array}$$

where ${\delta }_{0}$ denotes the coherence length of the partially coherent beams, *H*_*m*_ denotes the Hermite polynomial of order *m*, ${L}_{n}^{0}$ denotes the Laguerre polynomial of mode order *n* and 0. Due to the statistical properties of the partially coherent beams and the random phase distributions, the resulting phase distributions shown in Fig. 2b, c are not unique under the abovementioned generation condition of PSD. Figure 2d shows an illustration of the spatial coherence manipulation with the disordered metasurface. When illuminating the metasurface at different instants of time and different positions, different instantaneous speckle distributions are acquired containing the spatial correlation information of the light beam. The coherence length and coherence structure can be obtained from the statistical superposition of the instantaneous intensity distributions at the source plane. The instantaneous speckle distributions can be obtained through different methods, such as mechanical scanning, piezoelectric effect, structured illumination.

### Coherence structure modulation and beam shaping by the metasurfaces

**Fig. 3:**
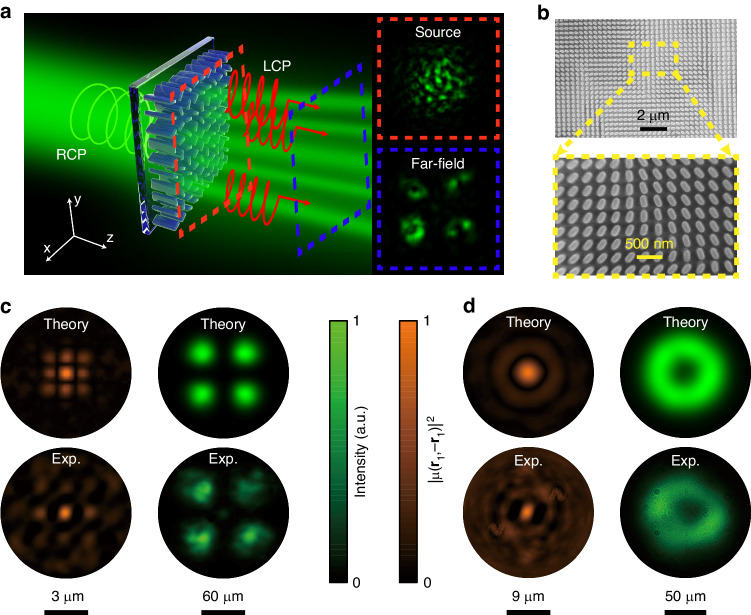
**Spatial coherence structures manipulated by the disordered metasurface and propagation properties of the generated beams. a** A right-handed circularly polarized incident light is focused on the metasurface, and the transmitted left-handed circularly polarized light can be collected by an objective paired with a tube lens. The propagation planes at different z_n_ are imaged by an imaging system to capture instantaneous intensity distributions at the source plane and far-fields. **b** SEM images of the fabricated metasurface. **c**, **d** The spatial coherence structure and far-field intensity distributions of the HG_22_-correlated (**c**) and LG_5_-correlated (**d**) beams customized in theory (top) and their measured counterparts (bottom)

Figure 3a illustrates the detailed experimental process for capturing the intensity distributions and measuring the coherence structure. The experimental setup is shown in Fig. S5 in Supplementary Material. The disordered metasurface is controlled by an electric translation stage to uniformly move along the *x*-direction for loading a phase change to the wave-front correlated with a designed DOC. A right-handed circularly polarized Gaussian beam with a beam size of 15 μm is illuminated on the sample. An imaging system combined with an objective ×100, a tube lens (TL), and a CCD camera is used to capture the transmitted cross-polarized light and record the light distributions to verify the spatial coherence manipulation of the incident fields. The disordered metasurface is fabricated with the standard atomic layer deposition method through single-step lithography^43,44^. The scanning electron micrograph (SEM) of the fabricated sample is shown in Fig. 3b. The size of the fabricated disordered metasurface is 240 × 40 μm (*x* × *y*). More details about the sample fabrication can be found in Methods. We obtained ×1000 images of the instantaneous speckle intensities, and the coherence manipulation is achieved by statistically combining numerous instantaneous speckle intensities of the beam cut-planes. The accuracy of the recovered DOC is related to the number of instantaneous intensity captures used for ensemble average (see Fig. S2 in Supplementary Material). The square of the modulus of DOC of the beams can be obtained by analyzing the statistical properties of the instantaneous speckle intensity distributions. Generally, the coherence structure of the partially coherent beams is defined at the source plane of the beams where the metasurface locates, after the propagation of the light beam, the coherence structure gradually degrades into a Gaussian distribution (see Fig. S6 in Supplementary Material). We calculated and measured the coherence structure at a short distance from the sample plane (*z* = 5 μm and 50 μm for HGCSM and LGCSM beams, respectively), to obtain a series of distinct speckle intensity distributions and the coherence-correlated speckles distributions of the instantaneous intensity. The theoretical and experimental spatial coherence structures manipulated by the metasurfaces are almost identical to the prescribed $\mu ({{\mathbf{\rho }}}_{2}-{{\mathbf{\rho }}}_{1})$, as shown in the left column of Fig. 3c,d.

The spatial coherence structure determines the light distribution of the beams in the far-field. Such spatially distributed fields can carry transportable optical information of the metasurfaces. As shown in the right columns of Fig. 3c,d, the partially coherent beam with HG_22_-correlated structure splits into four light spots, while the beam with LG_5_-correlated structure possesses a dark hollow in the center. Both of the coherence structure and the far-field optical information can be arbitrarily shaped owing to the one-to-one correspondence between the DOC and far-field light distribution. The coherence structure can decide the beam profile in the far field. The coherence length can also affect the intensity distributions of these beams in the far field and the robustness of these beams during propagation. The detailed propagation properties of these beams with different coherence structures and coherence lengths are discussed in Supplementary Note S1.

### Self-reconstruction of the HG_01_-correlated Schell-model beams with the metasurface

**Fig. 4:**
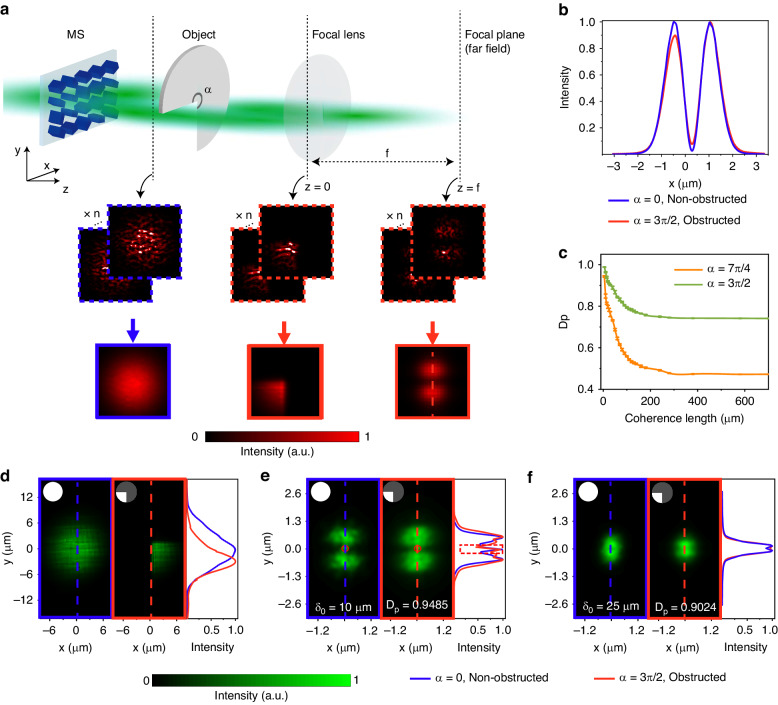
**Experimental self-reconstruction of the HG**_**01**_**-correlated beam blocked by a sector-shaped opaque object. a** Schematic of the self-reconstruction and intensity distributions of the partially coherent beams manipulated by the metasurface. **b** The theoretical cut-lines of the intensity distributions of the obstructed and unobstructed beams. **c** *D*_*p*_ as a function of the coherence length of the HG_01_-correlated Schell-model beams obstructed by different sector-shaped opaque objects. **d** Intensity distributions for the unobstructed and α = 3/2π obstructed optical transmission at the *z* = 0 plane. **e**, **f** Intensity distributions of the beams at the focal plane for unobstructed and α = 3/2π obstructed optical transmission with a coherence length of 10 μm (**e**) and 25 μm (**f**)

We further experimentally demonstrate the self-reconstruction of the HG_01_-correlated Schell-model beams with coherence lengths of δ_0_ = 10 μm and δ_0_ = 25 μm (Fig. 4). The schematic diagram of the beam self-reconstruction is shown in Fig. 4a. An obstacle placed away from the metasurface is illuminated by the generated partially coherent beam with a specially correlated coherence structure. A lens is used to perform the Fourier transform of the partially coherent beams to obtain the intensity at an infinite distance according to the Fraunhofer diffraction theorem. To better characterize the optical information carried by the beams, we normalized the light intensity to its maximum for both of the obstructed and unobstructed beams to exclude the effect of energy loss. The calculated intensity distributions of the obstructed and unobstructed beams are consistent with each other (Fig. 4b). The slight deviation is caused by the loss of a small portion of the coherence structure. Such information loss can be further decreased if decreasing the coherence length. The spatial coherence structure induces a re-arrangement of the energy distribution of the partially coherent beam as the beam propagates. Since the coherence structure is a global effect that is encoded to the whole beam, the partially coherent beam possesses self-similarity even for large obstructed angle α. We employed the self-reconstruction similarity degree (*D*_*p*_) to characterize the similarity of the two intensity distributions and the self-reconstruction capability, which can be expressed as${D}_{p}=\frac{{[\iint \langle {I}_{wt}({\mathbf{\rho }})\rangle \langle {I}_{ob}({\mathbf{\rho }})\rangle {d}^{2}{\mathbf{\rho }}]}^{2}}{\iint \langle {I}_{wt}({\mathbf{\rho }})\rangle {d}^{2}{\mathbf{\rho }}\iint \langle {I}_{ob}({\mathbf{\rho }})\rangle {d}^{2}{\mathbf{\rho }}}$. We theoretically calculated *D*_*p*_ as a function of the coherence length of the HG_01_-correlated Schell-model beams obstructed by an α = 3/2π and 7/4π sector-shaped opaque object, respectively (Fig. 4c). It is demonstrated that the self-reconstruction capability is independent of the obstacle shape in Fig. S8 in Supplemental Material. The *D*_*p*_ increases as the coherence length decreasing and reaches almost the same when ${\delta }_{0}\to 0$. Figure 4d shows the measured beams without and with an α = 3/2π sector-shaped opaque object at the *z* = 0 plane. And more measured self-reconstructed beams can be found in Fig. S9 in Supplemental Material. The optical information at $z\to \infty$ is measured at the focal plane with different coherence lengths (Fig. 4e,f). Compared with the unobstructed beam intensities, the obstructed ones rampantly excluding 3/4 areas of the beam can still provide sufficient optical information. The self-construction performance with δ_0_ = 10 μm is better than that with δ_0_ = 25 μm, and the calculated *D*_*p*_ are 0.9485 and 0.9024, respectively. Consequently, the coherence length and coherence structure of the partially coherent beams both determine the self-reconstruction ability. The presented self-reconstruction effect of the partially coherent beams can be used for robust information transfer and optical communications.

### Self-reconstructed imaging with the disordered metasurfaces

**Fig. 5:**
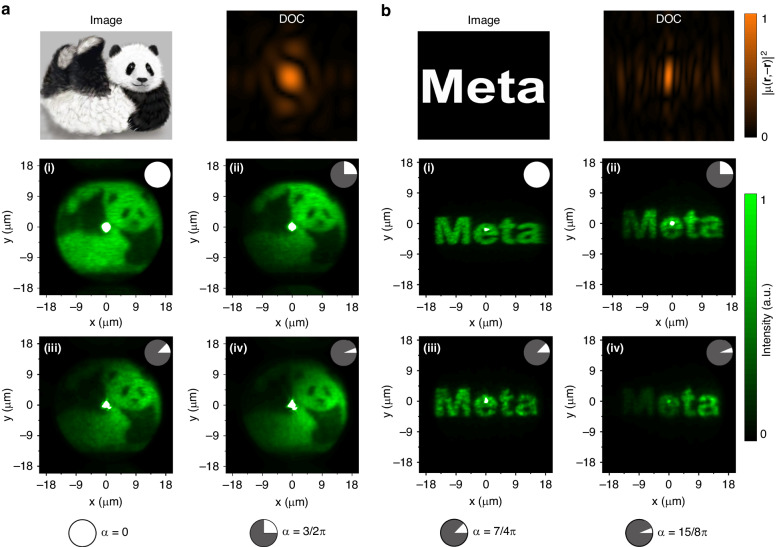
**Self-reconstructed imaging by metasurface based on spatial coherence structures engineering.** The target image ‘Panda’ (**a**) and ‘Meta’ (**b**) to design the metasurface and the corresponding DOC distribution (top). (i)-(iv): the intensity distributions at the focal plane with different obstacles

We further demonstrate the proposed strategy also enables self-reconstructed imaging with the designed disordered metasurfaces (Fig. 5). We fabricated two disordered metasurfaces with different DOCs related to two target images ‘Panda’ and ‘Meta’, as shown in the top left panels of Fig. 5a,b. The measured coherence structure information stored in the metasurfaces is shown in the top right panels. Compared with the images without any obstacles [(i) of Fig. 5a,b], all of the setups with different obstructed angles provides significant far-field self-reconstruction of the images [(ii)-(iv) of Fig. 5a,b]. The bright dot in the center is caused by the zero-order light resulting from the imperfection of fabrication and measurement, which more or less incorporates unwished polarized light. Surprisingly, even if the angle of the sector-shaped opaque object is as large as 15/8π, the details of the images are still well preserved. Especially shown in Fig. 5a, the characteristic information of the small-sized eyes and nose of the panda can still be retained. Meanwhile, the overall field-of-view (FOV) information of the patterns is also completely preserved, demonstrating the robustness of both the detailed and global optical information. The intensity loss near the boundary of the FOV is caused by the experimental error such as deviation of the FOV measured in the experiment, which is caused by the imperfect modulation of the collimation of the optical devices.

## Discussion

The proposed scheme not only simplifies the spatial coherence manipulation setup but also significantly improves the compactness and performance. Compared with conventional methods to manipulate the degree of spatial coherence such as the combination of the rotating ground glass disk and SLM, our strategy provides more accurate manipulation of speckle distributions with high efficiency due to the subwavelength nature. Compared with conventional informational strategies such as holography, which also enables global information encoded to the localized areas, our strategy provides robustness against scatterers and disturbance during light transmission owing to the statistical property. Note that the ratio of obstructed light can be further increased even approaching 100%, as long as the DOC is sufficiently small and the signal-to-noise ratio is experimentally guaranteed. Compared with conventional diffractive optical elements (DOE), the disordered metasurfaces can enable higher efficiency and resolution in information transmission, shorter coherence length and faster convergence during the wave propagation, which benefit further applications in integrated optical manipulation and coherence steering at micro-nanoscale. The resolution of the reconstructed image is closely related to the coherence length of partially coherent beams. If the coherence length is sufficiently low, the resolution is mainly limited by the diffraction limit. Moreover, the design strategy to manipulate the spatial coherence proposed here is not only limited to the specific beams mentioned in this work. Arbitrary Schell-model light beams can be generated by the metasurface, which can carry different information by their spatial coherence structures. Our approach can also be potentially expanded to carry dynamic information taking advantage of the versatile local optical resonances of metasurfaces, which may enable multiplexing of serial ensemble averages^19^. We can further improve the imaging speed with fast cameras, and the scanning process can be readily improved to kilohertz with up-to-date technology such as using the micro-electro mechanical system and structured lighting. Furthermore, the proposed strategy can also be extended to multi-wavelength metasurfaces, enabling robust full-color imaging capabilities in complex environments^45^.

In summary, we have proposed a strategy to simultaneously manipulate the spatial coherence structure and coherence length of the light beams based on disordered metasurfaces. By loading a random wave-front with a specific correlation function, the proposed metasurface can accurately manipulate the incident beams with a predefined spatial coherence structure and coherence length, and the experimental results are consistent with the theoretical calculations. Our strategy enables a wide branch of partially coherent beams with arbitrarily designed coherence lengths, such as HGCSM beams and LGCSM beams. We have demonstrated the robust optical information transmission and self-reconstruction with the disordered metasurface even if most of the light is recklessly obstructed during light transmission, which might shed new light on optical information transmission in disordered and perturbative media. Our scheme provides a generic principle for the generalized coherence manipulation and paves the way towards a plethora of applications in robust holography, optical computation and beam steering.

## Materials and methods

### Sample fabrication

The dielectric disordered TiO_2_ metasurfaces were manufactured on a meticulously prepared fused silica substrate. Initially, a 550 nm layer of electron-beam resist (specifically, ZEP 520 A by Zeon) was uniformly applied to the substrate using a spin-coating process. This coated substrate was then subjected to a hot plate treatment at 180 °C for a duration of 1 min. To prevent any electric charge buildup during the subsequent electron-beam writing step, a thin layer of E-spacer 300Z (Showa Denko) was applied atop the resist layer. The precise nanostructures were defined through electron-beam lithography, specifically employing a Raith150 instrument operating at 30 kV with a current of 20 pA and a dose of 80 μC/cm^2^. Subsequently, the exposed structures were developed in an n-amyl acetate solvent at room temperature for a period of 60 s. A conformal layer of TiO_2_, with an approximate thickness of 70 nm, was deposited onto the substrate using an atomic layer deposition system (Picosun). This deposition process involved the use of titanium tetrachloride and H_2_O as precursor materials within a reactor maintained at a temperature of 130 °C. Following the deposition, a blank-etching procedure of the TiO_2_ layer was carried out using CHF_3_ plasma within an inductively coupled plasma-reactive ion etching (ICP-RIE) system. Under controlled conditions, the TiO_2_ layer was etched until the underlying ZEP 520 A resist was exposed. These etching conditions involved the use of 20 sccm of CHF_3_, with a bias power of 20 W and an induction power of 500 W, all within an operating pressure of 10 mTorr. This process resulted in an etching rate of approximately 40 nm per minute for the TiO_2_ layer. Finally, O_2_ plasma with a minor addition of CHF_3_ was used to fully remove the remaining ZEP resist.

### Supplementary information

Supplementary Material

## Data Availability

The data that support the finding of this study are available from the corresponding author upon request.
