# Supplementary material for: Ultra-robust informational metasurfaces based on spatial coherence structures engineering
Source: Light Sci Appl. 2024 Jun 4;13:131. doi: 10.1038/s41377-024-01485-3 (PMC11150268; doi:10.1038/s41377-024-01485-3)
Supplement: Supplementary file 1 — Supplementary Material [file 41377_2024_1485_MOESM1_ESM.docx]

**Supplemental Material for “****Ultra-Robust Informational Metasurfaces Based on Spatial Coherence Structures Engineering”**

**Leixin Liu^1^, Wenwei Liu^2*^, Fei Wang^4^, Xiaofeng Peng^1^, Duk-Yong Choi^3^, Hua Cheng^2^, Yangjian Cai^1*^, Shuqi Chen^2,5*^**

*^1^Shandong Provincial Engineering and Technical Center of Light Manipulations, Collaborative Innovation Center of Light Manipulation and Applications, Shandong Provincial Key Laboratory of Optics and Photonic Device, School of Physics and Electronics, Shandong Normal University, Jinan 250014, China*

*^2^The Key Laboratory of Weak Light Nonlinear Photonics, Ministry of Education, Smart Sensing Interdisciplinary Science Center, School of Physics and TEDA Institute of Applied Physics, Nankai University, Tianjin 300071, China*

*^3^Laser Physics Centre, Research School of Physics, Australian National University, Canberra, ACT 2601, Australia*

*^4^School of Physical Science and Technology, Soochow University, Suzhou 215006, China*

*^5^The Collaborative Innovation Center of Extreme Optics, Shanxi University, Taiyuan, Shanxi 030006, China*

**Corresponding authors, email:* [*wliu@nankai.edu.cn*](mailto:wliu@nankai.edu.cn)*; yangjian_cai@163.com;* [*schen@nankai.edu.cn*](mailto:schen@nankai.edu.cn)

**SUPPLEMENTAL FIGURES**





**FIG. S1. Calculated comparison among the pure phase screens and the complex screens**. **a**,**b** The amplitude (**a**) and phase (**b**) of the complex screen. **c**,**d** The degree of the spatial coherence (**c**) and intensity distribution (**d**) of the light transmitted from the complex screen. **e**,**f** The amplitude (**e**) and phase(**f**) of the pure phase screen. **g**,**h** The degree of the spatial coherence (**g**) and intensity distribution (**h**) of the light transmitted from the pure phase screen.

**
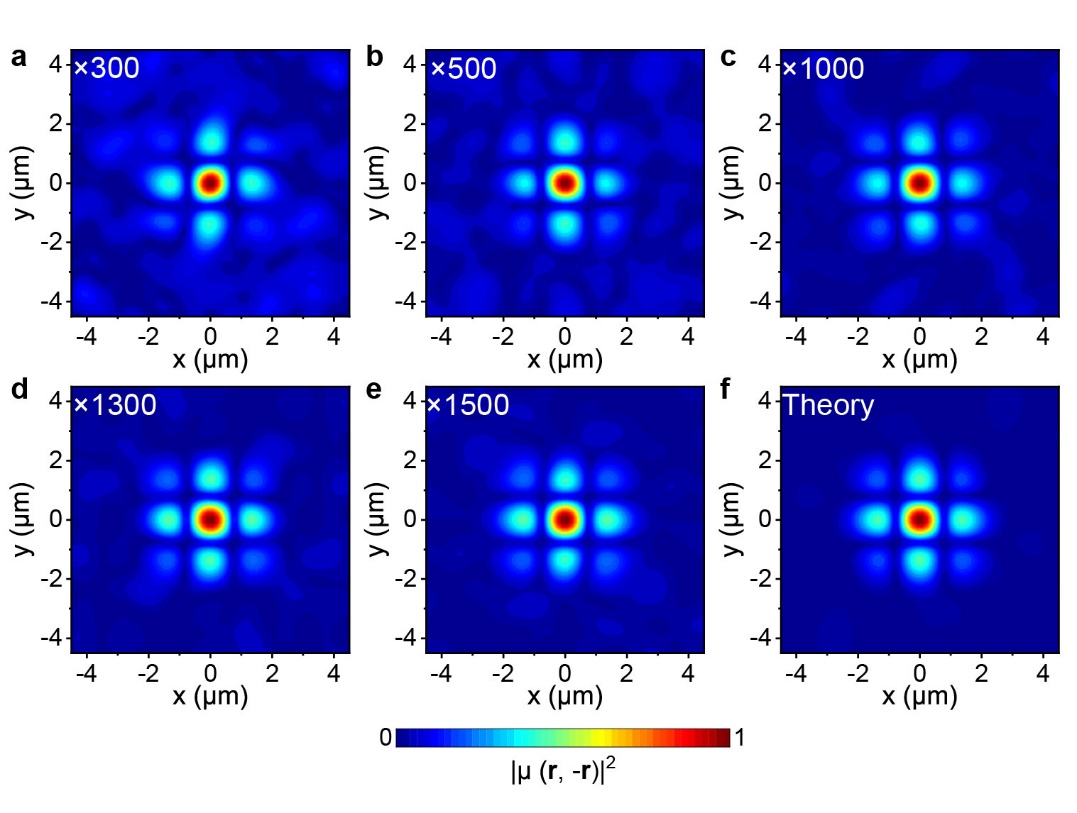
**

**FIG. S2. a-e**, Simulated spatial coherence of the HG_11_ correlated beams with 10 μm coherence length by computing the spatial correlation with (**a**) ×300, (**b**) ×500, (**c**) ×1000, (**d**) ×1300, and (**e**) ×1500 captures of instantaneous speckle intensities at the source plane. **f**. Theoretical spatial coherence of the HG_11_ correlated beams with 10 μm coherence length.


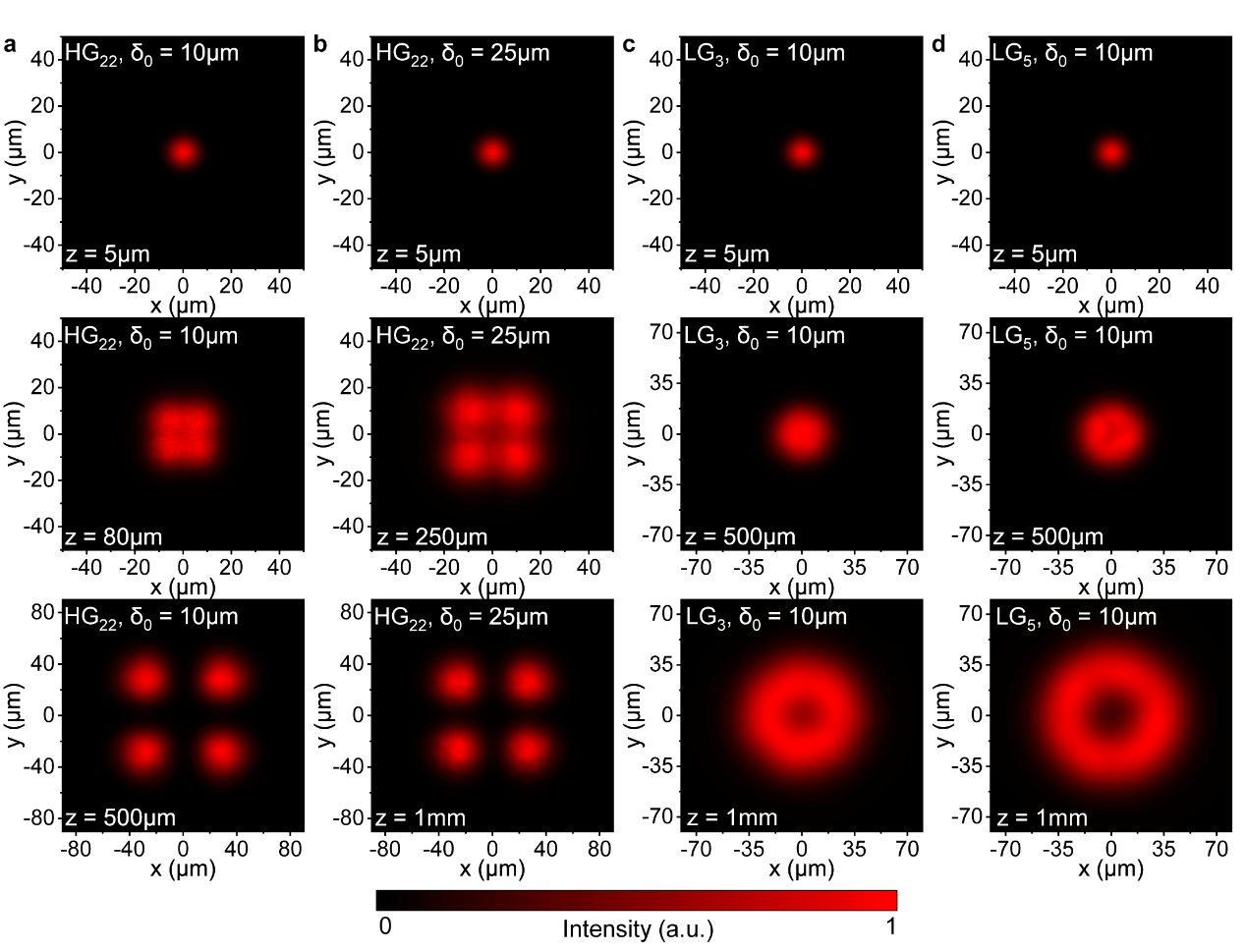


**FIG. S3. Propagation properties of the beams manipulated by the disordered metasurface in theory.** Theoretical intensity distributions of the beams with different coherence structure and coherence length, with (**a**) HG_22_ correlated Schell-model beams with 10 μm coherence length, (**b**) HG_22_ correlated Schell-model beams with 25 μm coherence length, (**c**) LG_3_ correlated Schell-model beams with 10 μm coherence length, and (**d**) LG_5_ correlated Schell-model beams with 10 μm coherence length, at different imaging distances from the source plane. The intensity distributions gradually converge at short distances, and the different propagation properties of these beams are induced by their corresponding spatial coherence distribution.

**

**

**FIG. S4. Propagation properties of the beams manipulated by the disordered metasurface in experiment.** Experimental intensity distributions of the beams with different coherence structure and coherence length, with (**a**) HG_22_ correlated Schell-model beams with 10 μm coherence length, (**b**) HG_22_ correlated Schell-model beams with 25 μm coherence length, (**c**) LG_3_ correlated Schell-model beams with 10 μm coherence length, and (**d**) LG_5_ correlated Schell-model beams with 10 μm coherence length, at different imaging distances from the source plane.


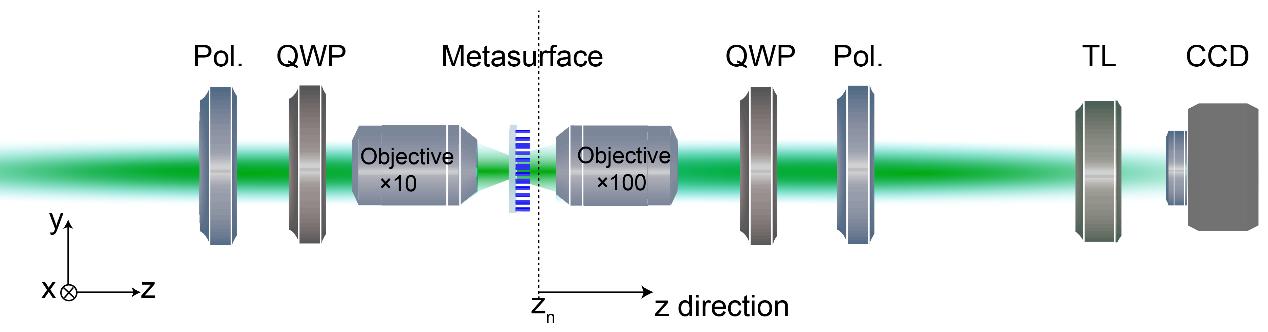


**FIG. S5. Experimental setup to capture the intensity distributions of the beams manipulated by the disordered metasurface.** A right-handed circularly polarized incident light is focused by a ×10 objective onto the metasurface, and the transmitted left-handed circularly polarized light is collected by a ×100 objective paired with a tube lens (TL). The source plane (*z* = 0) and propagation planes at different z_n_ are imaged by the imaging system.

**
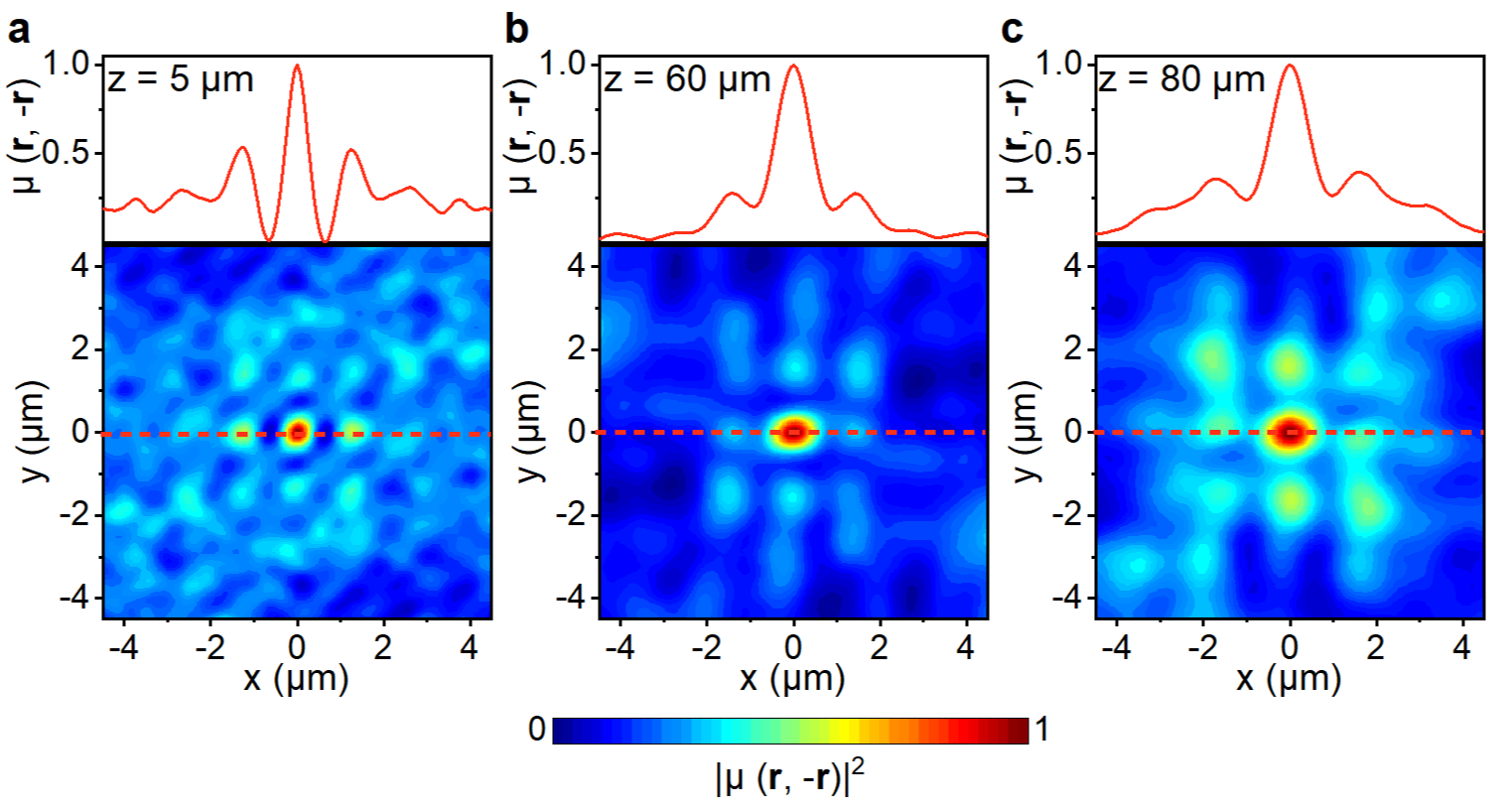
**

**FIG. S6. Measured spatial coherence of the HG_22_ correlated beams with 10 μm coherence length at different propagation distances z.** Experimental distributions and *y* = 0 cut-lines of the square of the degree of the coherence |μ(**r**, −**r**)|^2^  of HG_22_ correlated Schell-model beams with 10 μm coherence length.


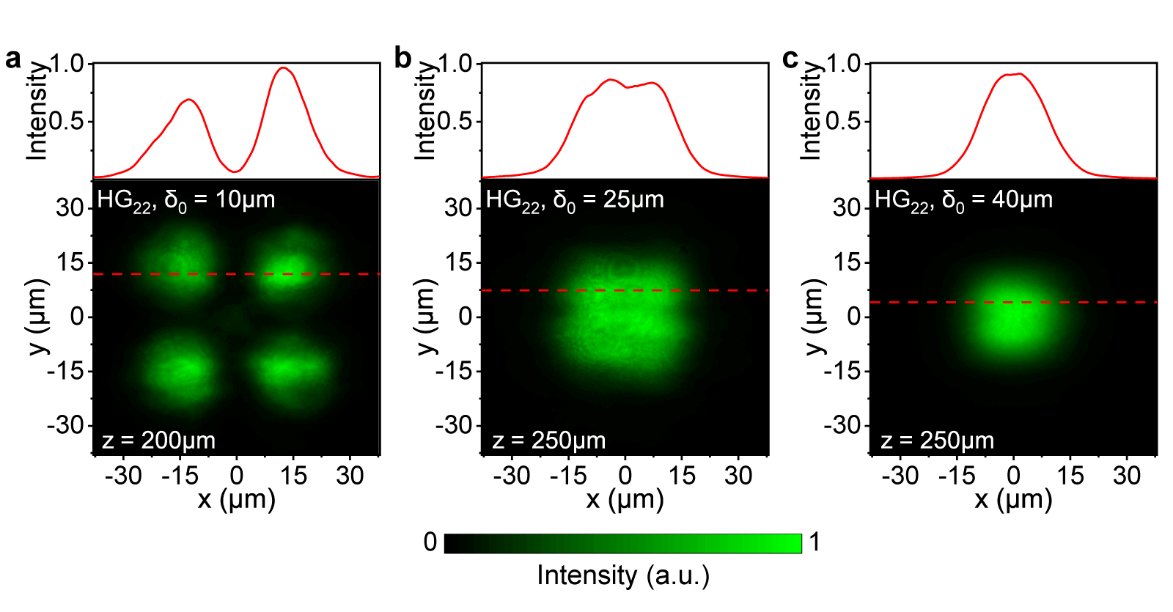


**FIG. S7. Propagation properties of the beams manipulated by the disordered metasurface with different coherence lengths.** Experimental intensity distributions of the beams with HG_22_ coherence structure and different coherence lengths. To compare the light distributions of different coherence, we selected corresponding off-center cut-lines of the intensity distributions at (**a**) *y* = 11.93 μm, (**b**) *y* = 7.41 μm, and (**c**) *y* = 4.16 μm, respectively.

**
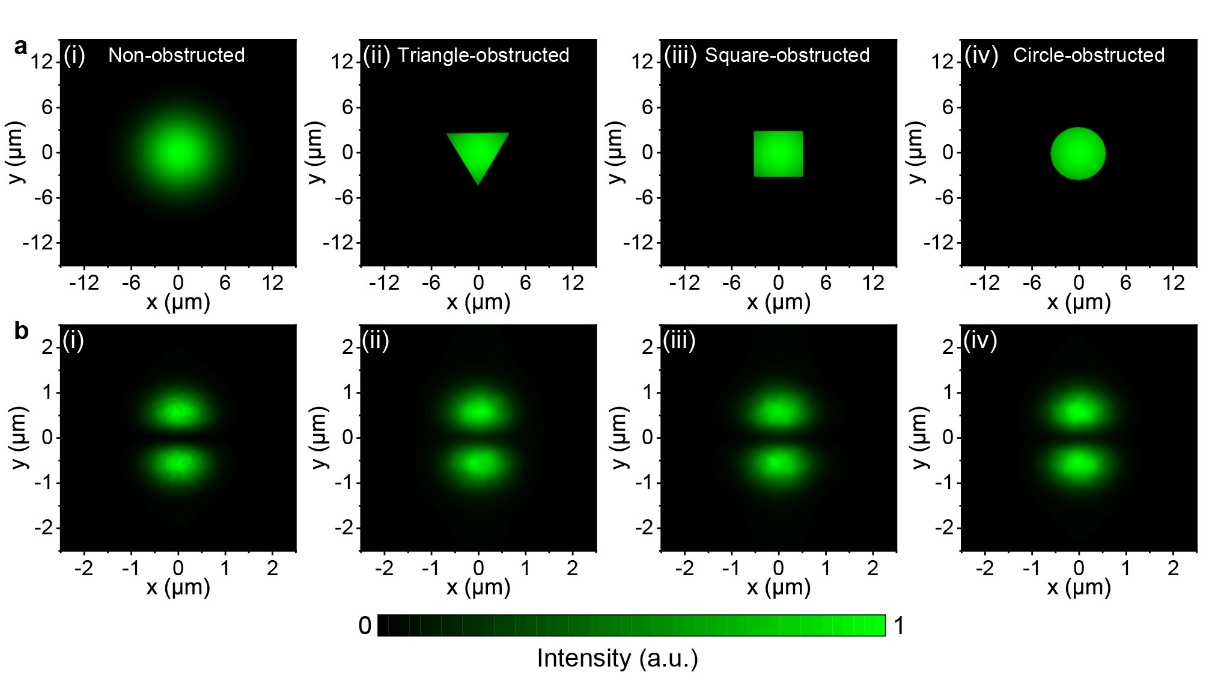
**

**FIG. S8. Self-reconstruction of the HG_01_ correlated Schell-model beams scattered by opaque object with different shape.** **a**. Intensity distributions for the unobstructed and different-shape-obstructed optical transmission at the z = 0 plane. **b**. Intensity distributions of the beams at the focal plane for unobstructed and obstructed optical transmission with a coherence length of 10 μm.


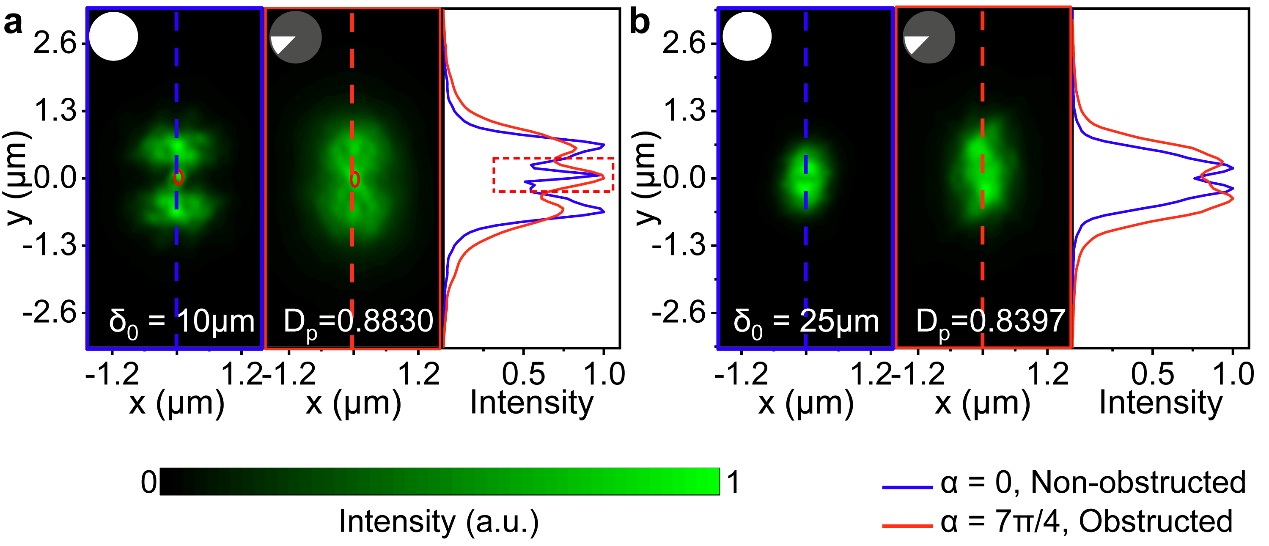


**FIG. S9. Experimental self-reconstruction ability of the HG_01_ correlated Schell-model beams scattered by a sector-shaped opaque object. a**,**b**, Intensity distributions at the focal plane with (**a**) a 10 μm coherence length and (**b**) a 25 μm coherence length. Measured intensity distributions for (Left) an unobstructed object, (middle) a 7/4π sector-shaped opaque object, and (right) corresponding cut-lines.

**SUPPLEMENTAL NOTE S1: THE PROPAGATION PROPERTIES OF THE DOC-MODULATED BEAMS.**

The average intensity distributions of the instantaneous intensities for HG_22_, LG_3_ and LG_5_ correlated Schell-model beams with different coherence are obtained at different image distances *z*_n_, which are the statistical average of the square of the modulus of fluctuating EM waves at point $\mathbf{\rho}=\mathbf{\rho}_{1}=\mathbf{\rho}_{2}$ in Eq. (2) (The theoretical intensity distributions of these beams are shown in Supplemental Figure S3). As the partially coherent beams propagate, the intensity profiles evolve from Gaussian distribution to different distributions for different coherent structures (Fig. S4). The partially coherent beams with HG_22_ coherence structure show a self-splitting property associated with the coherence lengths of the HGCSM beams. The lower the coherence length, the faster the HGCSM beam splits to meet a convergence during wave propagation (Fig. S7). In contrast, the LGCSM beams with *n* = 5 and *n* = 3 whose coherence lengths are both δ_0_ = 10 μm show a dark hollow during propagation, i.e., a ring-shaped beam profile in the far-field. When increasing the order of the LGCSM beam, the size of the dark hollow also increases. The experimental results of the HGCSM beams and LGCSM beams are both consistent with the theoretical results shown in Supplemental Figure S3.

**SUPPLEMENTAL NOTE S2: MEASUREMENT PROCEDURE**

As schematically depicted in Supplemental Figure S5, the laser beam generated by a supercontinuous laser (NKT SuperK EXR-20) is collimated by a fiber collimator. Using a polarizer combined with a quarter-wave plate, the metasurface is illuminated by a right-handed circularly polarized light. The first objective (10×) was used to limit the beam width smaller than each region of the metasurface. The movement of the disordered metasurface along the *x*-direction was controlled by an electric translation stage (e-X). Before measurement, the disordered metasurface and e-X need to be calibrated by a three-dimensional translation stage and a rotation stage. The optical fields with a generated coherence structure was collected by another objective (×100), and filtered by a quarter-wave plate and polarizer pair to obtain the left-handed circularly polarized component of the signals. The instantaneous intensity of the transmitted light was then captured by a tube lens (TL) and a CCD camera. In the experiments, we measured the light distributions at various planes with different *z*_n_ to obtain the evolution of the coherence structures and lengths. For the measurement of light intensity distributions at the infinite distance, a convex lens was employed behind the TL.
